# Significance of the orexinergic system in modulating stress-related responses in an animal model of post-traumatic stress disorder

**DOI:** 10.1038/s41398-020-0698-9

**Published:** 2020-01-21

**Authors:** Shlomi Cohen, Michael A. Matar, Ella Vainer, Joseph Zohar, Zeev Kaplan, Hagit Cohen

**Affiliations:** 1 Ministry of Health, Beer-Sheva Mental Health Center, Anxiety and Stress Research Unit, Faculty of Health Sciences, Ben-Gurion University of the Negev, Beer- Sheva, Israel; 2 Department of Psychology, Ben-Gurion University of the Negev, Beer-Sheva, Israel; 3 Division of Psychiatry, The Chaim Sheba Medical Center, Ramat-Gan, Israel; 4 Sackler Medical School, Tel-Aviv University, Tel-Aviv, Israel

**Keywords:** Molecular neuroscience, Psychiatric disorders

## Abstract

Converging evidence indicates that orexins (ORXs), the regulatory neuropeptides, are implicated in anxiety- and depression-related behaviors via the modulation of neuroendocrine, serotonergic, and noradrenergic systems. This study evaluated the role of the orexinergic system in stress-associated physiological responses in a controlled prospective animal model. The pattern and time course of activation of hypothalamic ORX neurons in response to predator-scent stress (PSS) were examined using c-Fos as a marker for neuronal activity. The relationship between the behavioral response pattern 7 days post-exposure and expressions of ORXs was evaluated. We also investigated the effects of intracerebroventricular microinfusion of ORX-A or almorexant (ORX-A/B receptor antagonist) on behavioral responses 7 days following PSS exposure. Hypothalamic levels of ORX-A, neuropeptide Y (NPY), and brain-derived neurotrophic factor (BDNF) were assessed. Compared with rats whose behaviors were extremely disrupted (post-traumatic stress disorder [PTSD]-phenotype), those whose behaviors were minimally selectively disrupted displayed significantly upregulated ORX-A and ORX-B levels in the hypothalamic nuclei. Intracerebroventricular microinfusion of ORX-A before PSS reduced the prevalence of the PTSD phenotype compared with that of artificial cerebrospinal fluid or almorexant, and rats treated with almorexant displayed a higher prevalence of the PTSD phenotype than did untreated rats. Activated ORX neurons led to upregulated expressions of BDNF and NPY, which might provide an additional regulatory mechanism for the modulation of adaptive stress responses. The study indicates that the activated ORX system might promote adaptive responses to PSS probably via stimulation of BDNF and NPY secretion, and early intervention with ORX-A reduces the prevalence of the PTSD phenotype and increases the prevalence of adaptive phenotypes. The findings provide some insights into the mechanisms underlying the involvement of the ORX system in stress-related disorders.

## Introduction

Orexins (ORXs; also called hypocretins), which are neuropeptides derived from the prepro-orexin precursor by post-translational proteolytic cleavage, are exclusively localized in the lateral and posterior hypothalamic nuclei^1,2^. Prepro-orexin is cleaved into two highly conserved peptides, ORX-A and ORX-B, which bind to two G-protein-coupled receptors, ORX1R and ORX2R, with different affinities^1,2^. ORX1R has a tenfold greater affinity for ORX-A than for ORX-B, whereas ORX2R has nearly equal affinity for both neuropeptides^2^. ORX neurons receive functional inputs from multiple systems distributed in the cortex, limbic system, and subcortical areas (including the hypothalamus and thalamus) and ascending projections from the brain stem cholinergic nuclei, reticular formation, midbrain raphe nuclei, and periaqueductal gray^1^. Therefore, these ORX neurons project throughout the central nervous system^3–7^. The afferent and efferent projections and the functional activity of ORX neurons suggest that ORXs play a role in neuroendocrine and behavioral responses, including arousal^3,6,8,9^, wakefulness, cognitive function, goal-oriented motor behaviors^10^, feeding and reward processes^11,12^, thermogenesis, energy metabolism, reproduction, development, aging, emotional memory^13^, secretion of hormones, autonomic responses, and stress responses^2,4,14,15^.

Behavioral, pharmacological, and genetic studies have investigated the relationship between the ORXergic system and anxiety-related behaviors and stress responses, and the findings indicate that the system is involved in stress regulation and coping. It has been demonstrated that in rats, microinjections of both ORX-A and ORX-B in the paraventricular nucleus of the midline thalamus (PVT) decrease exploration in the open field but increase grooming and freezing, which are behavioral indicators of an aversive state^16^, suggesting that ORX release in the PVT enhances negative emotional behaviors^16^. In addition, stressful conditions tend to alter ORXergic-dependent awakening processes^15,17^. Therefore, the upregulation of the ORX system be implicated in the maintenance of high arousal and anxiety-like behavior. However, low baseline plasma and cerebrospinal fluid (CSF) ORX-A levels have been observed in combat veterans with post-traumatic stress disorder (PTSD), and ORX-A levels are negatively correlated with PTSD severity^18^. Our recent study also indicates that anxiolytic effects of modafinil (a wakefulness-promoting drug) following traumatic stress exposure are mediated by the upregulation of hypothalamic ORX levels^19^. These contradictory findings indicate that the association between the altered ORX system and behavioral responses to fear events remains to be investigated^20^. Notably, Martins et al.^21^ showed that ORX-A levels increased in the CSF after acute forced swim stress but decreased following long-term immobilizations in rats, and the findings indicate that acute stress may be associated with increased ORX activity/levels, whereas chronic stress could lead to low ORX activity/levels^15^. Altogether, these studies demonstrate the possible involvement of ORXs in the pathophysiology of PTSD, and the findings provide a rationale for studying the role of the ORXergic system in animal models of PTSD.

Because accumulating evidence demonstrates that Fos protein is expressed in neurons whose activity is strongly stimulated by synaptic inputs, the expression of the immediate early gene *(c-fos)* is widely considered a high-resolution marker of neuronal activity^22^. In addition, previous studies have suggested that the ORXergic system could regulate the secretion of brain-derived neurotrophic factor (BDNF)^23,24^ and neuropeptide Y (NPY)^25,26^, which provide greater resiliency to stress by increasing synaptic plasticity and stabilizing synaptic connectivity^27,28^. In this study, our working hypothesis was that the ORXergic system is actively involved in the neurobiological response to predator-scent stress (PSS), and an early intervention with ORX-A thus reduces the prevalence of the PTSD phenotype and increases the prevalence of adaptive phenotypes compared to that with artificial cerebrospinal fluid (ACSF) and ORX antagonist (almorexant). We also hypothesized that the ORXergic system promotes adaptive responses to stress, in part via stimulation of BDNF and NPY secretion. To this end, we first examined the expressions of ORX-A, ORX-B, and c-Fos in the paraventricular nucleus (PVN) of the hypothalamus and lateral hypothalamus (LH) before PSS exposure and 60, 90, 120, 150, or 300 min following PSS exposure to investigate molecular changes immediately following PSS. Second, we examined whether a single exposure to PSS has a long-term effect on protein expression levels of ORX-A and ORX-B in the PVN and LH in rats. Third, a controlled, prospective trial was conducted to examine the effects of an ORX agonist or antagonist, which was administered 30 min before stress exposure, on behavioral stress responses, and the expressions of BDNF and NPY in the PVN.

## Materials and methods

All treatment and testing procedures were approved by the Animal Care Committee of Ben-Gurion University of the Negev, Israel.

### Animals

A total of 112 adult male Sprague-Dawley rats, weighing 180–220 g, were used in this study. The rats were housed (three per cage) in a vivarium with a stable temperature under a 12:12-h light–dark cycle (lights off at 19:00 hours; luminous emittance during the light phase: 200G50 lux), with unlimited access to food and water. All rats were allowed for a 1-week habituation period before the experiment. All procedures were performed during the resting phase of the rats between 08:30 and 12:00.

### Experimental design

We conducted three experiments to assess the effects of orexinergic activity in an established rat model at various time points along the PTSD trajectory. The experimental design used for each of these experiments is schematically depicted in the respective figures. In experiment 1 (*N* = 31), which aimed to elucidate the molecular changes associated with PSS, rats were exposed to PSS for 15 min. The expressions of ORX-A, ORX-B, and c-Fos in the hypothalamus before PSS exposure or 60, 90, 120, 150, or 300 min following PSS exposure were evaluated. In Experiment 2, rats (*N* = 38) were exposed to PSS/sham PSS for 15 min. Behaviors were assessed on day 7 using the elevated plus maze (EPM) and the acoustic startle response (ASR) assay. The prevalence rates of extreme, partial, and minimal behavioral responses (EBR, PBR, and MBR) were assessed in rats exposed to PSS/sham PSS. On day 8, rats were sacrificed and their brains (*N* = 21) were collected for immunoreactivity analyses of ORX-A and ORX-B in the hypothalamic nuclei. Experiment 3 (*N* = 43) evaluated the effects of pharmacological manipulations [ORX-A (ORXs agonist) vs. ACSF and almorexant (ORXs antagonist) vs. saline] of the endogenous ORXergic system prior to PSS exposure on behavioral responses. Behaviors were assessed on day 7, and rats were sacrificed and their brains were collected on day 8.

### Predator-scent stress

Rats were individually placed on a well-soiled cat litter, which had been used by a cat for 2 days and sifted for stools. The rats were exposed to the litter for 10 min in a plastic cage (inescapable exposure) on a concrete paving stone in a closed environment. Sham PSS was administered under similar conditions, but the rats were exposed to a fresh, unused cat litter^29–32^.

### Drugs

Orexin-A, SB674042 ([5-(2-fluorophenyl)β-2-methyl-4-thiazolyl] [2(S)-2-[(5-phenyl-1,3,4-oxadiazol-2-yl) methyl-1-pyrrolidinyl] methanone]), was purchased from Sigma-Aldrich (Israel). ORX-A was dissolved in ACSF (Sigma-Aldrich, Israel) on the day of the experiment and was administered via intracerebroventricular (ICV) microinjection (AP: −0.4 mm; ML: ±1.3 mm; DV: −4.2 mm relative to bregma)^33^.

*Almorexant,* (2R)-2-[(1S)-6,7-dimethoxy-1-[2-(4-trifluoromethylphenyl)-ethyl]-3,4-dihydro-1H-isoquinolin-2-yl]-*N*-methyl-2-phenyl-acetamide (150 mg kg^−1^), was synthesized at ChemBo Pharma (Kowloon, Hong Kong). Almorexant was dissolved in saline and was injected intraperitoneally^19^.

### Behavioral measurements

Behaviors of rats were assessed using EPM and ASR, as described previously^27,34^. The detailed protocols are described in Supplementary Information 1.

### Cut-off behavioral criteria model

Individual rats were classified according to the degrees to which individual behavior was affected by a stressor. The classification of individual rats was based on the premise that extremely compromised behavior in response to the priming trigger is not conducive to survival, is inadequate and maladaptive, and thus represents a pathological degree of response^29–32^. Please see Supplementary Information 1 for a detailed explanation of the criteria.

### Immunohistochemistry

Please see Supplementary Information 1 for a detailed description of the methods used for immunohistochemistry.

### Statistical analyses

For the results of molecular changes, statistical analyses were performed using one-way analysis of variance (ANOVA). Bonferroni tests were used to examine differences between individual groups. In addition, behavioral data were transformed into percentages using the cut-off behavioral criteria (CBC) model. The prevalence of affected rats was examined using cross-tabulation and nonparametric chi-squared tests. All nonparametric analyses were performed using raw data (and not on the percentages). Pearson's correlation analysis was used to determine the relationship between behavioral variables and brain ORX levels in the entire sample.

Two rates were calculated for ORX-A and ORX-B in order to obtain a standard measurement scale for ORX-A and ORX-B in all brain areas, and each was based on the mean of the standardized *Z*-scores^35^. ORX-A and ORX-B *Z*-scores were individually calculated for each PSS-exposed rat (*N* = 17), irrespective of behavioral assessments. An unsupervised k-means cluster analysis was conducted using STATISTICA 12 based on ORX-A and ORX-B *Z*-scores (Dell Inc., USA).

For the behavioral effects of pharmacological manipulation of ORXs levels, statistical analyses were performed using a two-way ANOVA with PSS exposure (PSS vs. Sham PSS) and treatment (ACSF/ORX-A/almorexant) as independent factors.

## Results

### Effects of PSS exposure on c-Fos and ORX expressions in the hypothalamus nuclei

**Fig. 1:**
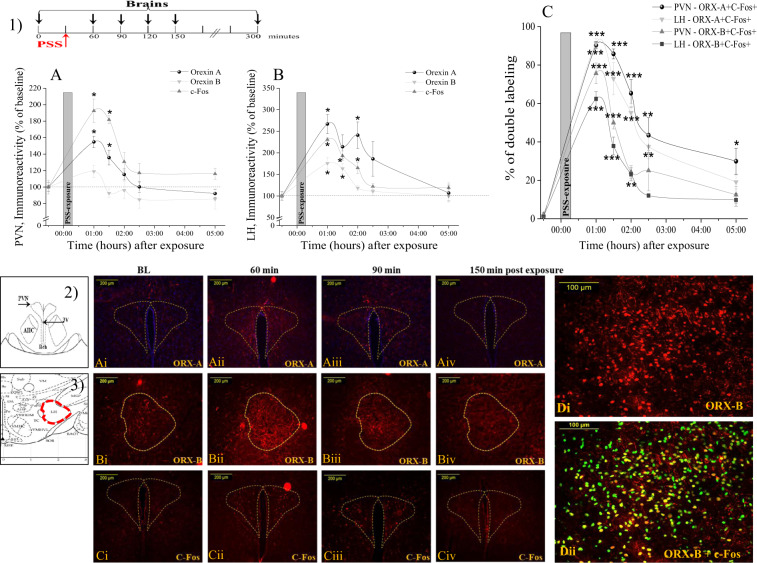
Effects of PSS exposure on c-Fos and ORX expressions in the hypothalamus nuclei. (1) The experimental protocol is depicted on the top panel. Animals were brought to the lab in their home cage and were exposed to PSS for 15 min. Brains were taken at 0 (baseline), and 60, 90, 120, 150, or 300 min following PSS exposure. A schematic drawing representing the PVN (2) and LH (3) regions from which measurements were collected. The quantitative morphometric analysis of ORX-A and ORX-B immunoreactivity (ir) in the fibers and cells and c-Fos activated neurons (% of baseline) in the PVN (**a**) and LH (**b**) for baseline controls (*n* = 6), 60 min (*n* = 5), 90 min (*n* = 5), 120 min (*n* = 5), 150 min (*n* = 5), and 300 min (*n* = 5) after PSS exposure. **c** Percentage of double-labeled neurons for Fos/ORX-A/B in the PVN and LH before and following PSS exposure. **a**i–iv Representative photomicrographs of ORX-A expression (red) in the PVN area before (**a**i), 60 (**a**ii), 90 (**a**iii), and 150 (**a**iv) min after PSS exposure (scale bar = 200 μm). **b**i–iv Representative photomicrographs of ORX-B expression (red) in the LH area before (**b**i), 60 (**b**ii), 90 (**b**iii), and 150 (**b**iv) min after PSS exposure (scale bar = 200 μm). **c**i–iv Representative photomicrographs of c-Fos expression (red) in the PVN area before (**c**i), 60 (**c**ii), 90 (**c**iii), and 150 (**c**iv) min after PSS exposure (scale bar = 200 μm). **d**i–ii Activated orexin-A neurons (**d**i) were identified by the presence of nuclear labeling in the c-Fos neurons (**d**ii) using double immunofluorescence staining in the LH 60 min post-exposure (scale bar = 100 μm). The double-label fluorescence protocol produced very similar results to those described above for the enzyme–substrate protocol, with c-Fos-ir fluorescing green and orexin-A-ir fluorescing res. A green fluorescing c-Fos-ir nucleus viewed through red fluorescing orexin-A-ir cytoplasm appeared as yellow. Because c-Fos and orexin-A are located in different cellular compartments, cells in the cutting plane exhibit a distinct green (c-Fos-ir) nucleus surrounded by red (orexin-A-ir) cytoplasm. There appeared to be no cross-reactivity between the c-Fos and orexin-A-ir. Data shown as the percentage of baseline level; bars represent group means ± S.E.M.; *p < 0.01, **p < 0.005, ***p < 0.0005 compared to their baseline levels. PSS predator-scent stress, LH lateral hypothalamus, ORX orexin, PVN paraventricular nucleus of hypothalamus. All data represent group mean ± S.E.M.

C-Fos-positive nuclei were very scarce in the PVN and LH in unexposed animals (baseline). In contrast, an increased number of c-Fos-positive nuclei was observed in the PVN and LH in rats exposed to PSS (Fig. 1). One-way ANOVA revealed a significant time-dependent effect of PSS exposure on the number of c-Fos-positive nuclei in the PVN [*F*(5, 25) = 17.8, *p* < 0.0001] and LH [*F*(5, 25) = 34.7, *p* < 0.0001]. Bonferroni post hoc tests confirmed a marked increase in the number of c-Fos-positive cells in the PVN 60 (*p* < 0.0001) and 90 min (*p* < 0.0001) following PSS exposure compared with baseline (Fig. 1a). Increased c-Fos levels in the LH were observed 60 min (*p* < 0.0001), 90 min (*p* < 0.0001), and 120 min (*p* < 0.0003) following PSS exposure compared with baseline (Fig. 1b). In both regions, c-Fos expressions gradually declined to the baseline level in approximately 150 min.

PSS exposure significantly increased the number of ORX-A-positive cells and fibers in the PVN and LH (Fig. 1). One-way ANOVA revealed a significant time-dependent effect of PSS exposure in the PVN [*F*(5, 25) = 13.23, *p* < 0.000] and LH [*F*(5, 25) = 7.4, *p* < 0.000]. Bonferroni post hoc tests confirmed a marked increase in the density of ORX-A-positive cells and fibers in the PVN 60 min (*p* < 0.0001) and 90 min (*p* < 0.01) following PSS exposure compared with baseline (Fig. 1a). Increased ORX-A levels in the LH were observed 60 min (*p* < 0.002) and 120 min (*p* < 0.008) following PSS exposure compared with baseline (Fig. 1b). In both regions, ORX-A levels gradually declined to the baseline level in approximately 6 h.

A significant time-dependent effect of PSS exposure on ORX-B expressions was observed only in the LH (*F*(5, 25) = 10.6, *p* < 0.000) (Fig. 1b). Increased ORX-B-positive cells and fibers in the LH were observed 60 min (*p* < 0.0002) and 90 min (*p* < 0.002) following PSS exposure compared with baseline. Fisher least significant difference (LSD) comparisons revealed significantly increased ORX-B levels in the PVN 60 min after PSS exposure compared with baseline (*p* < 0.04). In both regions, elevated ORX-B levels gradually declined to the baseline level in approximately 6 h.

Double-label immunohistochemistry for c-Fos and ORXs was performed on brain sections to identify activated ORXs neurons. Percent changes of c-Fos-positive cells to ORX-A/B neurons in the PVN and LH before and after PSS exposure are shown in Fig. 1c. As expected, both the PVN and LH were virtually devoid of c-Fos+/ORXs+ neurons in the baseline condition. In contrast, robust activation of c-Fos in ORX^+^ cells and fibers was observed 1 h after PSS exposure. In the PVN and LH areas, percentages of c-Fos-positive cells to ORX-A-positive cells were maximal at 1 h post PSS exposure (c-Fos labeling occurred in 90.3% and 91.3% of ORX-A- and ORX-B-positive cells, respectively) and then declined gradually. In the PVN, 5 h after PSS, the number of c-Fos^+^/ORX-A^+^ neurons was decreased but did not recover completely. In the PVN and LH, 2 h after PSS, the number of c-Fos^+^/ORX-B^+^ neurons was decreased to the baseline level. Taken together, these results suggest that endogenous ORXs are activated in response to PSS. Activation of these neurons may be associated with increased vigilance, a trait associated with anxiety^15^.

### Behavioral disruption following PSS is associated with altered long-term ORX expressions

**Fig. 2:**
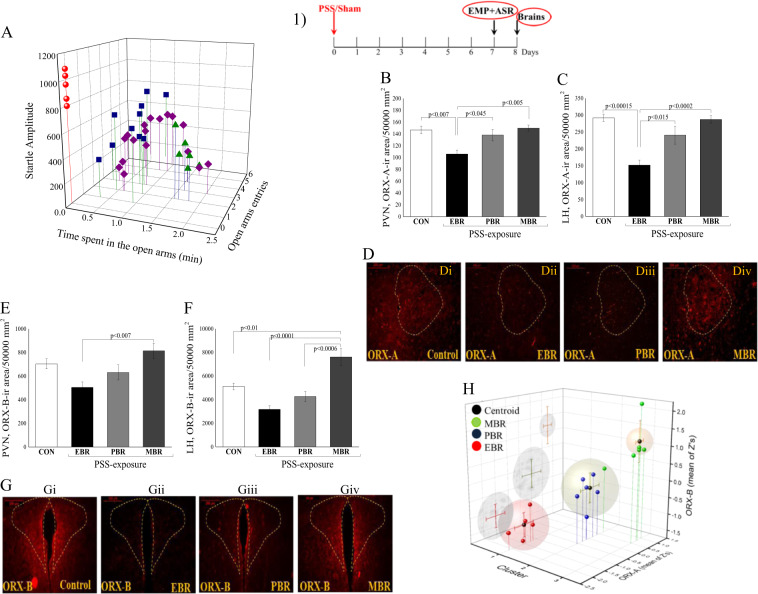
Behavioral disruption following PSS is associated with altered long-term ORX expressions. (1) The experimental protocol is depicted on the top panel. Rats were exposed for 15 min to predator-scent stress (PSS) or to sham PSS on day 0, tested in the elevated plus maze (EPM) and acoustic startle response (ASR) on day 7 and sacrificed on day 8 for brain removal and analysis. **a** Effect of the PSS on overall anxiety-like behavior and startle response: Diamonds (purple) represent the unexposed control group. Circles (red) represent the exposed group that exhibited a significant degree of anxiety-like and avoidant behaviors on the elevated plus maze, and a pattern of exaggerated startle responses with significantly reduced habituation 7 days after exposure (EBR, PTSD phenotype). Squares (blue) represent the exposed group that exhibited partial behavioral response patterns (PBR). Triangles (green) represent the exposed group that exhibited minimal behavioral disruption (MBR). Three-dimensional parameters: the *X*-axis represents time spent in the open arms (min), *Y*-axis represents acoustic startle amplitude, and *Z*-axis represents startle habituation. The stress response was not homogeneous, and several subgroups were identifiable in the population. The quantitative morphometric analysis of ORX-A immunoreactivity (ir) in the fibers and cells in area/50,000 mm^2^) in the PVN (**a**) and LH (**b**) 8 days post PSS exposure in unexposed controls (CON, *n* = 17), in animals with extreme behavioral response (EBR, *n* = 5), or partial response (PBR, *n* = 10) to the PSS exposure, and in animals whose behavior was minimally affected by the stressor (MBR, *n* = 6). **d**i–iv Representative photomicrographs of ORX-A expression (red) in the LH in unexposed controls (**d**i), in animals with extreme behavioral response (**d**ii), or partial response (**d**iii) to the PSS exposure, and in animals whose behavior was minimally affected by the stressor (**d**iv). In the PVN and LH regions, EBR animals exhibited significantly lower ORX-A-ir levels than did unexposed rats and MBR rats. The quantitative morphometric analysis of ORX-B immunoreactivity (ir) in the fibers and cells in area/50,000 mm^2^) in the PVN (**e**) and LH (**f**) 8 days post PSS exposure in unexposed controls (CON, *n* = 17), in animals with extreme behavioral response (EBR, *n* = 5), or partial response (PBR, *n* = 10) to the PSS exposure, and in animals whose behavior was minimally affected by the stressor (MBR, *n* = 6). **g**i–iv Representative photomicrographs of ORX-B expression (red) in the LH in unexposed controls (**g**i), in animals with extreme behavioral response (**g**ii), or partial response (**g**iii) to the PSS exposure, and in animals whose behavior was minimally affected by the stressor (**g**iv). In the PVN and LH regions, EBR animals exhibited significantly lower ORX-B-ir levels compared to MBR rats. **h** Unsupervised K-means cluster analysis: Unsupervised K-means cluster analysis, based on two means of *Z*-score transformed data, for ORX-A and ORX-B, which were calculated for each rat. Black dots represent analysis centroids extracted from data. Colored dots represent the original CBC classification of each rat, EBRs (red), PBRs (blue), and MBRs (green). As the analysis indicates, unsupervised clustering classified rats into three distinctive groups, based only upon ORX-A and ORX-B *Z*-scores. Classification was 94% (16/17 cases) similar to behavioral classification as performed using the CBC algorithm. Horizontal and vertical bars represents group ORX-A-ir and ORX-B-ir S.E., respectively. ASR acoustic startle response, CON control, EBR extreme behavioral response, EPM elevated plus maze, MBR minimal behavioral response, ORX orexin, PBR partial behavioral response, PSS predator-scent stress, PVN paraventricular nucleus of hypothalamus. All data represent group mean ± S.E.M.

A broad range of variations in behavioral responses was observed in PSS-exposed animals, and several subgroups were thus identified (Fig. 2a). Accordingly, the animals were subdivided into groups reflecting the magnitudes of responses (including EBR, PBR, and MBR) according to the CBCs. Approximately 23.8% of the exposed animals (*N* = 5/21) fulfilled criteria for PTSD phenotype (EBR); 28.6% (*N* = 6/21) for MBR; and 47.6% for PBR (*N* = 10/21).

#### ORX-A

Significant differences in ORX-A levels were found among the groups in the PVN (Fig. 2b) and LH (Fig. 2c) areas [one-way ANOVA: *F*(3, 19) =6.92, *p* < 0.005 and *F*(3, 19) =13.18, *p* < 0.0001, respectively]. Bonferroni post hoc tests confirmed significantly lower ORX-A-positive cells and fibers in the PVN and LH areas in EBR (*p* < 0.007 and *p* < 0.005, respectively), MBR (*p* < 0.005 and *p* < 0.005, respectively), and PBR animals (*p* < 0.05 in both areas) compared with unexposed controls. In the hypothalamic regions, no significant differences were found among MBR, PBR, and unexposed groups.

#### ORX-B

There were significant differences in ORX-B levels among the groups in the PVN (Fig. 2e) and LH (Fig. 2f) areas [one-way ANOVA: *F*(3, 19) = 5.17, *p* < 0.01 and *F*(3, 19) = 14.88, *p* < 0.0005, respectively]. Bonferroni post hoc tests confirmed significantly lower ORX-B-positive cells and fibers in the PVN and LH areas in the EBR group than in the MBR group (*p* < 0.007 and *p* < 0.0001, respectively). In addition, significantly higher ORX-B levels were found in the LH area in the MBR group than in the unexposed (*p* < 0.01) and PBR (*p* < 0.0006) groups. These findings indicate that ORX expressions in the hypothalamic nuclei 8 days following PSS exposure are associated with the degrees of behavioral responses.

### Correlation between altered ORX expressions and behavioral assessments

We further conducted regression analyses to gain additional understanding about the relationship between behavioral response patterns (8 days) and activated ORX levels in the hypothalamus, irrespective of the CBC classification. Pearson’s correlation analysis revealed that the time spent in the open arms was significantly correlated with ORX-A expressions in the LH (*r* = 0.74, *p* < 0.01) and ORX-B expressions in the PVN (*r* = 0.51, *p* < 0.05) and LH (*r* = 0.63, *p* < 0.01). Furthermore, the number of open arms entries was significantly correlated with ORX-A expressions in the PVN (*r* = 0.62, *p* < 0.05). The anxiety index was significantly correlated with ORX-A expressions in the PVN (*r* = −51, *p* < 0.056) and LH (*r* = −0.48, *p* < 0.05) and ORX-B expressions in the PVN (*r* = −0.57, *p* < 0.05) and LH (*r* = −0.54, *p* < 0.05). The startle amplitude was significantly correlated with ORX-A expressions in the PVN (*r* = −61, *p* < 0.01) and LH (*r* = −0.63, *p* < 0.01) and ORX-B expressions in the PVN (*r* = −0.61, *p* < 0.01).

In order to validate the close relations observed between the degrees of post-exposure behavioral disruptions and hypothalamus ORX neurons, molecular data of PSS-exposed rats (*N* = 17) were further analyzed using an unsupervised k-means cluster analysis. As shown in Fig. 2h, the unsupervised cluster analysis of mean *Z*-scores for ORX-A and ORX-B could identify three significantly different clusters, which defined three different ORX patterns, among PSS-exposed rats [ORX-A: *F*(2, 14) = 53.48, *p* < 0.0000; ORX-B: *F*(2, 14) = 31.58, *p* < 0.0000]. Further analyses of the case classifications by clusters according to their ORX-A and ORX-B *Z*-scores revealed 94% compatibility between the two independent classifications (goodness of fit *r*^2^ = 0.33, *p* < 0.85). Specifically, based on CBC, rats in cluster-1 were all classified as having EBRs; rats in cluster-2, except one MBR rat, were all classified as having PBRs; and rats in cluster-3 were all classified as having MBRs. Altogether, unsupervised clustering based upon ORX levels could be used to predict the behavioral phenotype classification, and the findings suggest the significance of orexin activity in recovery from a highly stressful experience.

### ORX-A intracerebroventricular microinjection prior to stress exposure attenuates subsequent behavioral stress responses

**Fig. 3:**
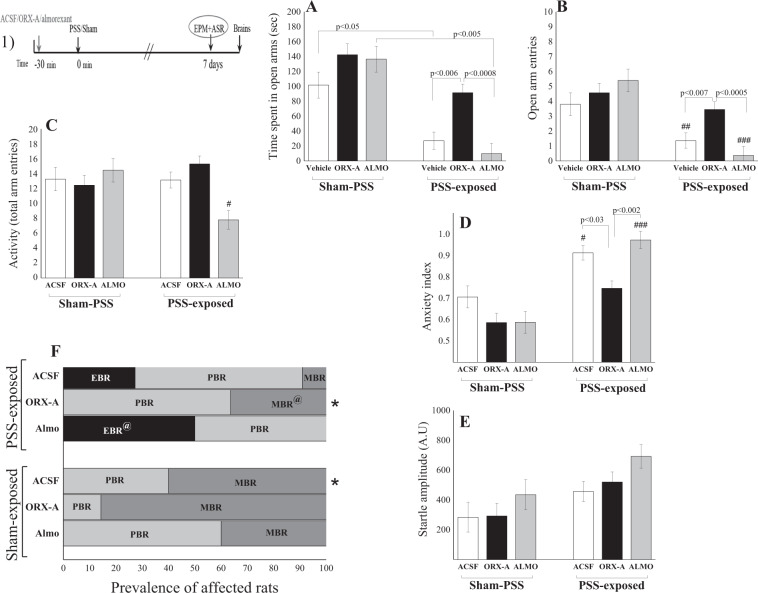
ORX-A intracerebroventricular microinjection prior to stress exposure attenuates subsequent behavioral stress responses. (1) The experimental protocol is depicted on the top panel. Rats received intracerebroventricular microinjection with either ORX-A (20 mg/kg; sham/ORX-A [*n* = 7]; PSS/ORX-A [*n* = 11]) or ACSF (2 µl; sham/ACSF [*n* = 5]; PSS-ACSF [*n* = 11]). In addition, two groups of rats were injected IP with almorexant, a dual OX_1_R/OX_2_R antagonist (150 mg/kg; sham/almorexant [*n* = 5]; PSS/almorexant [*n* = 8]) or vehicle (Saline) (sham/saline [*n* = 5]; PSS-Saline [*n* = 5]). No differences were observed for saline treatment and ACSF treatment for both sham PSS and PSS exposure and, therefore, the two groups were combined together into one control group. Behavioral measurements (EPM and ASR) were performed on day 7. **a** Time spent in the open arms of the EPM. **b** Number of entries to the open arms of the EPM. **c** Overall activity in the EPM, as reflected in the total number of entries to the open and closed arms. **d** Overall anxiety index, which integrates the measured behavioral measures on the EPM. **e** Startle amplitude in the ASR. Prevalence of CBC distribution for all tested groups; **f** The prevalence of extreme behavioral response (EBR) vs. partial behavioral response (PBR) and minimal behavioral response (MBR) rats (in percentages). Microinfusion of ORX-A prior to PSS exposure, significantly decreases anxiety-like behaviors, relative to ACSF microinfusion. Concomitantly, the prevalence of EBRs following PSS exposure is markedly lower in rats treated with ORX-A than in those treated with vehicle or almorexant. ^#^*p* < 0.05, ^##^*p* < 0.005, ^###^*p* < 0.0005 compared to correspondent sham PSS group; *p < 0.05 distribution is significantly different from PSS-ACSF group. ^@^*p* < 0.055 Fischer exact cross-tabulation analysis PSS-ORX-A vs. PSS-almorexant. ACSF artificial cerebrospinal fluid, ALMO almorexant, CON control, EBR extreme behavioral response, ASR acoustic startle response, EPM elevated plus maze, MBR minimal behavioral response, PBR partial behavioral, ORX orexin, PSS predator-scent stress. Bars represent group mean ± S.E.M. or percentages.

No differences in EPM or ASR were observed between saline treatment and ACSF treatment after both sham PSS and PSS exposure; therefore, we combined the control groups together (vehicle). Altered ORX-A levels prior to PSS exposure significantly affected behavioral variables in the EPM, including time spent in open arms [two-way ANOVA; Exposure (main effect): *F*(1, 41) = 49.88, *p* < 0.0001; Exposure × Treatment interaction: *F*(2, 41) = 3.48, *p* < 0.05], number of entries to open arms [two-way ANOVA; Exposure (main effect): *F*(1, 41) = 29.87, *p* < 0.0001; Exposure × Treatment interaction: *F*(2, 41) = 4.75, *p* < 0.02], and anxiety index [two-way ANOVA; Exposure (main effect): *F*(1, 41) = 50.80, *p* < 0.0001; Exposure × Treatment interaction: *F*(2, 41) = 3.65, *p* < 0.04] (Fig. 3). Rats microinfused with ORX-A prior to PSS exposure spent significantly more time in the open arms of the maze (Fig. 3a), showed more entries to open arms (Fig. 3b), and exhibited a significantly lower anxiety index (Fig. 3d) than did those exposed to PSS and treated with ACSF (Bonferroni test: *p* < 0.006, *p* < 0.007, and *p* < 0.03, respectively) or almorexant (Bonferroni test: *p* < 0.0008, *p* < 0.0005, and *p* < 0.002, respectively), indicating that microinfusion of ORX-A ameliorates behavioral effects of PSS exposure. Neither upregulation nor downregulation of ORX levels affected behavioral measurements 7 days following sham PSS treatment.

Significant differences in the prevalence rates of individual rats displaying extreme, partial, or minimal responses were found among the groups (Pearson *χ*^2^ = 25.94, df = 10, *p* < 0.004). Significant differences were observed in the prevalence rates of rats displaying EBR among the groups (Pearson *χ*^2^ = 14.0, df = 5, *p* < 0.02; Fig. 3f). The prevalence of EBR rats among PSS-exposed rats injected with almorexant was 50.0% of the total rats and differed significantly from that among PSS-exposed rats treated with ORX-A (Fisher exact *χ*^2^ = 6.97, *p* < 0.02), in which there were no EBR rats. There were significant differences in the prevalence rates of individual rats displaying MBR among the groups (Pearson *χ*^2^ = 17.1, df = 5 *p* < 0.0045; Fig. 3g). The prevalence rates of MBR among the PSS-exposed rats treated with ACSF, ORX-A, and almorexant were respectively 60%, 85%, and 40% of the total rats and differed significantly from those of unexposed animals (*χ*^2^ = 4.75, *p* < 0.03; *χ*^2^ = 4.22, *p* < 0.04; and *χ*^2^ = 3. 8, *p* < 0.05, respectively). The prevalence of MBR among PSS-exposed rats microinjected with ORX-A was 36.4% of the total rats and differed from that among PSS-exposed animals treated with almorexant (*χ*^2^ = 3.7, *p* = 0.055), in which there were no MBR rats. Altogether, these findings indicate that high levels of ORX-A before PSS lead to a significant shift toward less extreme behavioral disruption, and ORX-A induces some degrees of resistance to the stress-related sequelae.

**Fig. 4:**
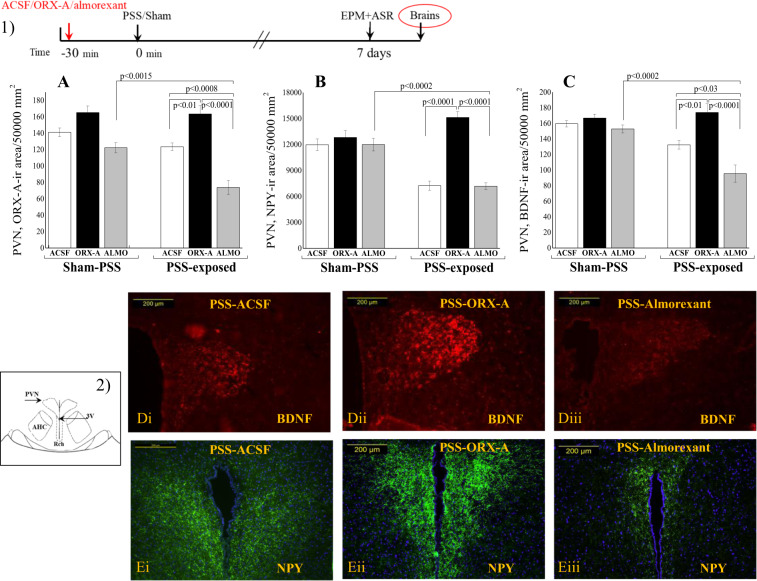
Molecular effects of ORX-A vs. ACSF and almorexant vs. saline injections before PSS or sham PSS exposure. (1) The experimental protocol is depicted on the top panel. Rats were microinjected ICV with either ORX-A (20 mg/kg; sham/ORX-A [*n* = 7]; PSS/ORX-A [*n* = 11]) or ACSF (2 µl; sham/ACSF [*n* = 5]; PSS-ACSF [*n* = 11]). In addition, two groups of rats were injected intraperitoneally with almorexant, a dual OX_1_R/OX_2_R antagonist (150 mg/kg; sham/almorexant [*n* = 5]; PSS/almorexant [*n* = 8]) or vehicle (Saline) (sham/saline [*n* = 5]; PSS-Saline [*n* = 5]). Behavioral measurements (EPM and ASR) were performed on day 7. The rats were sacrificed one day later (day 8) and their brains were collected for measurement of ORX-A, NPY, and BDNF immunoreactivity. **a** The quantitative morphometric analysis of ORX-A immunoreactivity (ir) in the fibers and cells of the PVN (two-way ANOVA: PSS: *F*(1, 30) = 13.9, *p* < 0.001, Treatment: *F*(2, 30) = 39.8, *p* < 0.0001, PSS–Treatment interaction: *F*(2, 30) = 5.1, *p* < 0.015). **b** The quantitative morphometric analysis of NPY-ir in the fibers and cells of the PVN (two-way ANOVA: PSS: *F*(1, 30) = 20.8, *p* < 0.0001, Treatment: *F*(2, 30) = 30.4, *p* < 0.0001, PSS-Treatment interaction: *F*(2, 30) = 19.9, *p* < 0.0001). **c** The quantitative morphometric analysis of BDNF-ir in the cells of the PVN (two-way ANOVA: PSS: *F*(1, 30) = 16.9, *p* < 0.0003, Treatment: *F*(2, 30) = 18.3, *p* < 0.0001, PSS–Treatment interaction: *F*(2, 30) = 8.9, *p* < 0.001). (2) A schematic drawing representing the PVN region from which measurements were collected. **d** The quantitative morphometric analysis of BDNF immunoreactivity (ir) (in area/50,000 mm^2^) in the PVN 8 days post PSS exposure in exposed rat treated with ACSF (**d**i), ORX-A (**d**ii), or almorexant (**d**iii). **e** The quantitative morphometric analysis of NPY immunoreactivity (ir) (in area/50,000 mm^2^) in the PVN 8 days post PSS exposure in exposed rat treated with ACSF (**e**i), ORX-A (**e**ii), or almorexant (**e**iii). ACSF artificial cerebrospinal fluid, ALMO almorexant, ASR acoustic startle response, BDNF brain-derived neurotrophic factor, EPM elevated plus maze, NPY neuropeptide Y, ORX orexin, PSS predator-scent stress, PVN paraventricular nucleus of hypothalamus. All data represent group mean ± S.E.M.

To elucidate the molecular mechanisms underlying these pharmacological changes, we examined the expressions of ORX-A, NPY, and BDNF in the PVN (Fig. 4a–c). Rats microinfused with ORX-A before PSS exhibited significantly higher ORX-A, NPY, and BDNF levels than did those microinfused with ACSF (*p* < 0.01, *p* < 0.001, and *p* < 0.01, respectively) or almorexant (*p* < 0.0001 for all groups).

## Discussion

The most significant findings of this study are as follows: (1) Orexin neurons in the PVN and LH hypothalamic nuclei were activated in response to PSS exposure. (2) There was a striking association between the degrees of behavioral disruption following PSS exposure and the patterns of changes in the orexinergic activity in the PVN and LH 8 days post-exposure. Greater activation of orexin neurons in the hypothalamic nuclei was correlated with minimal response phenotypes (MBR) and lesser activation of orexin neurons in the hypothalamic nuclei was correlated with PTSD phenotype (EBR). (3) The blockade of the ORX receptor exacerbated the behavioral effects of PSS, indicating a protective role of the endogenous ORXergic system. (4) Centrally administered ORX-A led to elevated endogenous BDNF and NPY expression, which might have contributed to the correction of the disruptive behavioral effects of PSS. These findings indicate that the orexinergic system plays an active role in the neurobiological response to PSS and may be related to the pathophysiology associated with PTSD, at least in some individuals.

We first studied the possible activation of ORX neurons in the LH and PVN hypothalamic nuclei immediately after PSS exposure. The pattern and time course of ORX activation in response to PSS exposure were examined using c-Fos expression as a marker to reflect the excitability of orexinergic neurons. Significant upregulated ORX-A and ORX-B levels in the LH and increased ORX-A levels in the PVN were observed immediately after PSS exposure compared with baselines. The stress-induced activation of hypothalamic ORX neurons has been demonstrated to increase arousal^36^ because ORX-containing neurons are critical components of the circuitry that regulates and determines the arousal threshold^9^, and the unique pattern of synaptic organization and plasticity of ORX neurons, in which excitatory (glutamatergic) contacts dominate inhibitory (GABAergic) ones, enables the ORXergic system to be easily and powerfully excited, thus inducing rapid arousal that leads to enhanced transmission of various auditory, olfactory, visual, and other sensory information (somatomotor, visceromotor, and hormonal)^14,37,38^. Arousal is the necessary initial response for an adequate (adaptive) behavioral response to stress^37,39^, in which animals maximize the functioning of their sensory systems to detect and respond to potential threats in the environment^14^. Therefore, we assume that increased ORX levels immediately after stress exposure enable the animal to evaluate the changing environment, prepare for potential threats, and respond in an effective manner.

In order to test this assumption, we first evaluated local brain levels of ORXs in stress-exposed animals 8 days after exposure, and subsequently, we manipulated ORX-A expressions by administering either antagonist (almorexant) or agonist in the second set of experiments. Both ORX-A and ORX-B levels in the hypothalamus were downregulated in animals whose behaviors were severely affected by the stressor (PTSD phenotype or EBR) but did not change in PBR, MBR, and control rats. Moreover, there was a striking negative correlation between the severity of behavioral disruption and changes in the central ORX expression. Pearson’s correlation analysis revealed that the downregulation in ORX levels was significantly correlated with the increased degree of anxiety-related behaviors, assessed by both EPM and ASR paradigms, across the entire sample. Our findings are supported by those in previous studies investigating ORX expressions in PTSD patients^18^. It has been demonstrated that CSF and plasma ORX-A levels are significantly lower in patients with PTSD than in healthy controls, and CSF ORX-A levels are strongly and negatively correlated with PTSD severity, as measured by the Clinician-Administered PTSD Scale, in patients with PTSD^18^. Taken together, these findings indicate that the ORXergic system is actively involved in the neurobiological response to traumatic stress and may be associated with resilience/recovery after the PSS exposure.

Unsupervised clustering analyses indicate that PTSD phenotype cases could be detected based upon distinct ORX levels at an accuracy rate of ~95%. Distinctive patterns in ORX activation have been attributed to the perceived characteristics of the stressor. Specifically, the inability to engage the threatening environment or react to the stressor results in inactivation of the orexinergic system^40,41^. In fact, in contrary to the upregulation of ORXs in response to acute stress, chronic stress has been shown to decrease ORXergic activity^12^. Notably, downregulated ORX levels are evident among major depression disorder (MDD) patients^42^. In humans, maximal ORX levels occur during social interactions and subject-reported positive emotions^13^, while conditions that are associated with minimal ORX levels can elicit withdrawal, such as pain and anxiety^43^.

The present study showed that a single dose of ORX-A microinfusion into the ventricular cavity 30 min before PSS exposure resulted in a significant moderation of behavioral patterns including stress-induced anxiety, avoidance, and hyperarousal responses in the EPM and ASR tests, and the findings support our assumption. Compared with ACSF treatment, ORX-A administration reduced prevalence rates of PTSD-like phenotypes (EBR) to nil and increased the prevalence of minimal and partial response patterns, i.e., a significant overall shift toward a more adaptive behavioral response to stress. In contrast, the pre-injection of almorexant, a dual orexin receptor antagonist, disrupted behavioral stress responses and significantly increased vulnerability to long-term anxiety-related behaviors. The aforementioned anxiolytic-like effect of ORX-A was accompanied by the significant upregulation of hypothalamic NPY and BDNF levels, which may increase synaptic plasticity and stabilize synaptic connectivity, leading to resilience. In contrast, almorexant treatment disrupted the expression of BDNF and NPY in the hypothalamus. Taken together, endogenous and/or exogenous activation of the ORXergic system enables animals to respond in an efficacious manner following stress exposure.

The ORXergic system is also involved indirectly in the response to stress through the activation of the hypothalamo-pituitary-adrenal (HPA) axis^39,44–47^. At the hypothalamic level, ORX may activate the HPA axis by increasing the release of CRF in the median eminence, ACTH, and corticosterone^44,46^. In addition, ORXs exert a selective and direct glucocorticoid secretagogue activity in the rat adrenal glands through a receptor-mediated activation of the adenylate cyclase/PKA-dependent signaling pathway^48–50^. Consequently, the activation of the HPA axis can further trigger ORX neuronal firing^14,46^. This cycle, in which ORX neurons play a role as both the trigger and accelerator, contributes to the activation and maintenance of arousal associated with the stress response^46^. The findings, which are in agreement with those in our previous studies, highlight the importance of an initial bolus of endogenous or exogenous corticosteroids in the adaptive response to stress and homeostasis^34,51–53^, and the increase in corticosterone levels following modafinil treatment (which upregulates endogenous hypothalamic ORXs shortly after administration) thus appears to contribute to an adaptive response^19^. Therefore, when the ORXergic system is activated, an array of coordinated peripheral organ and neuroendocrine responses arise, thus providing complementary support for adaptive behaviors^14,37,38^.

Our findings tend to support the notion that the ORXergic system promotes resilience via direct and active effects on the neurobiological response to stress. We hypothesize that in response to stress, when the ORXergic system is activated, ORX neurons can initiate (and maintain) behavioral stress responses by activating arousal, sensory, somatomotor, visceromotor, hormonal, and other systems, enabling the animals to better prepare for, respond to, and cope with the acute demands of physical and emotional threats to re-establish homeostasis. Mechanistically, our findings suggest that the ORXergic system stimulates circuits that link together NPY and BDNF, which have been shown independently to participate in adaptive stress responses, within the hypothalamus.

Notably, cognitive theories indicate that attention allocation to threats is disrupted in patients with PTSD^54,55^. Indeed, a tendency for attention to fluctuating between threat vigilance and threat avoidance, called “attention bias variability,” reliably correlates with PTSD symptoms^54–56^, which may reflect a loss of attentional control and aberrant buffering of attention among individuals with PTSD symptoms^55,57,58^. Inattention to threatening stimuli during acute stress increases the risk of PTSD^59,60^. Moreover, four sessions of attention bias modification training, delivered prior to combat deployment, mitigate PTSD risk following combat exposure^61^. Taken together, it is reasonable to hypothesize that the activated ORXergic system in the perceived threatening environment plays a role in attention bias towards threatening stimuli, enabling online vigilance and alertness to the threat and facilitates subsequent (“off-line”) recovery. In addition, failure or dysregulation of the ORXergic system during or following stress exposure may affect this attention bias, reduce alertness to potential threats, and then contribute to psychopathologies associated with traumatic stress. Further studies are needed to establish the connection between orexinergic activity and threat attention.

## Conclusions

ORXs orchestrate various aspects of survival behaviors in response to PSS. The appropriate activation of the ORXergic system in response to stress can not only cause a subject to better prepare for, respond to, and cope with the acute demands for physical and emotional stressors but also facilitate faster recovery from the threat. Our findings indicate that the ORXergic system plays an active role in the PSS response cascade and interacts with other systems including the HPA axis, BDNF, and NPYergic systems to initiate, coordinate, and maintain processes involved in adaptive stress-related behavioral responses. Therefore, in response to stress, the activated ORXergic system might stimulate endogenous activation of BDNF and NPYergic systems and provide an additional regulatory mechanism for the modulation of adaptive stress responses. Our findings suggest that the ORXergic system is related to the pathophysiology associated with PTSD. Further studies are needed to determine whether the ORXergic system is a possible therapeutic target for the treatment of stress-related disorders.

## Supplementary information

Supplemental Material
