# Supplementary material for: Significance of the orexinergic system in modulating stress-related responses in an animal model of post-traumatic stress disorder
Source: Transl Psychiatry. 2020 Jan 21;10:10. doi: 10.1038/s41398-020-0698-9 (PMC7026175; doi:10.1038/s41398-020-0698-9)
Supplement: Supplementary file 1 — Supplemental Material [file 41398_2020_698_MOESM1_ESM.docx]

**SUPPLEMENTARY 1**

**BEHAVIORAL MEASUREMENTS**

***Elevated plus-maze (EPM):***

The maze was a plus-shaped platform with two opposing open arms and two opposing closed arms (closed arms surrounded by 14-cm-high opaque walls on three sides) (12). Rats were placed on the central platform, facing an open arm, and were allowed to freely explore the maze for 5 min. Each test was videotaped and the behavior of the rat was subsequently scored by an independent observer. The arm entry was defined as entering an arm with all four paws. At the end of the 5-min test period, the rat was removed from the maze, the floor was wiped with a damp cloth, and any fecal boluses were removed. Behaviors assessed were time spent (duration) in open and closed arms; number of entries to the open and closed arms; and total exploration, which was calculated as the number of entries into any of the arms and was used to distinguish between impaired exploratory behavior, exploration limited to closed arms (avoidance), and free exploration. Finally, an “Anxiety Index,” which integrates the EPM behavioral measures, was calculated as follows:

$$\begin{matrix} Anxiety & Index=1-\left[\frac{\left(\frac{\begin{matrix} \begin{matrix} \begin{matrix} time & spent \end{matrix} & in & the \end{matrix} & open & arms \end{matrix}}{\begin{matrix} \begin{matrix} total & time & on \end{matrix} & the & maze \end{matrix}}\right)+}{\begin{matrix} & \begin{matrix} \begin{matrix} \begin{matrix} \begin{matrix} \begin{matrix} \begin{matrix} & & \end{matrix} & & \end{matrix} & & \end{matrix} & & \end{matrix} & & \end{matrix} & 2 \end{matrix} \end{matrix}}\frac{\left(\frac{\begin{matrix} \begin{matrix} \begin{matrix} number & of \end{matrix} & entries & to \end{matrix} & the & \begin{matrix} open & arms \end{matrix} \end{matrix}}{\begin{matrix} \begin{matrix} total & \exp loration & on \end{matrix} & the & maze \end{matrix}}\right)}{}\right] \end{matrix}$$

***Acoustic startle response (ASR):***

Startle responses were measured by using two ventilated startle chambers (SR-LAB system, San-Diego Instruments, San-Diego, CA, USA). The SR-LAB calibration unit was used routinely to ensure consistent stabilimeter sensitivity between the test chambers and over time. Each Plexiglas cylinder rested on a platform inside a soundproof, ventilated chamber. Movement inside the cylinder was detected by a piezoelectric accelerometer below the frame. Sound levels within each test chamber were measured routinely with a sound level meter (Radio Shack) to ensure consistent presentation. Each test session began with a 5-min acclimatization period to 68-dB background white noise, followed by 30 acoustic startle trial stimuli presented in 6 blocks (110 dB white noise of 40 ms duration and 30 or 45 s inter-trial interval). The mean startle amplitude (averaged over all 30 trials) was assessed.

***The Cut-off Behavioral Criteria (CBC) model:***

Human responses to traumatic experiences clearly vary greatly in extent and in character. More importantly, PTSD occurs in a percentage (roughly 25–35%) of the individuals exposed to potentially traumatic events, underscoring the importance of contemporary definitions of stress-related disorders (DSM and ICD) and of inclusion and exclusion criteria applied in controlled clinical trials. In contrast, most animal studies have tended to relate to unclassified “global” groups, i.e., the entire exposed population versus control populations without distinction, whereas researchers who work with animals have long been aware that individual study subjects tend to display a variable range of responses to stimuli, certainly where stress paradigms are concerned. The heterogeneity in animal responses might be regarded as confirming the validity of animal studies, rather than as a problem. It stands to reason that a model of diagnostic criteria for psychiatric disorders can be applied to animal responses to augment the validity of study data, as long as the criteria for classification are clearly defined, reliably reproducible, and yield results that conform to findings in human subjects. The criteria used in this model were thus based on the EPM and ASR paradigms combined, and they clearly define two opposing extremes of the possible responses of each individual to stress. The one extreme of this model, termed here an ‘extreme behavioral response’ (EBR), indicates animals whose exploration of the open arms of the EPM was zero throughout the test, and whose startle response was maximal and did not undergo any habituation throughout the ASR test. An EBR thus parallels extreme PTSD-like responses and un-abating maximal stress. The other extreme, termed here a ‘minimal behavioral response’ (MBR), indicates animals whose behavior in both the EPM and ASR paradigms was virtually unaffected by the stressor. A MBR thus parallels no PTSD-like response to the stressor. Rats that did not meet the criteria for either an EBR or a MBR were considered, by default, to have a partial behavioral response (PBR)^1-3^.

**
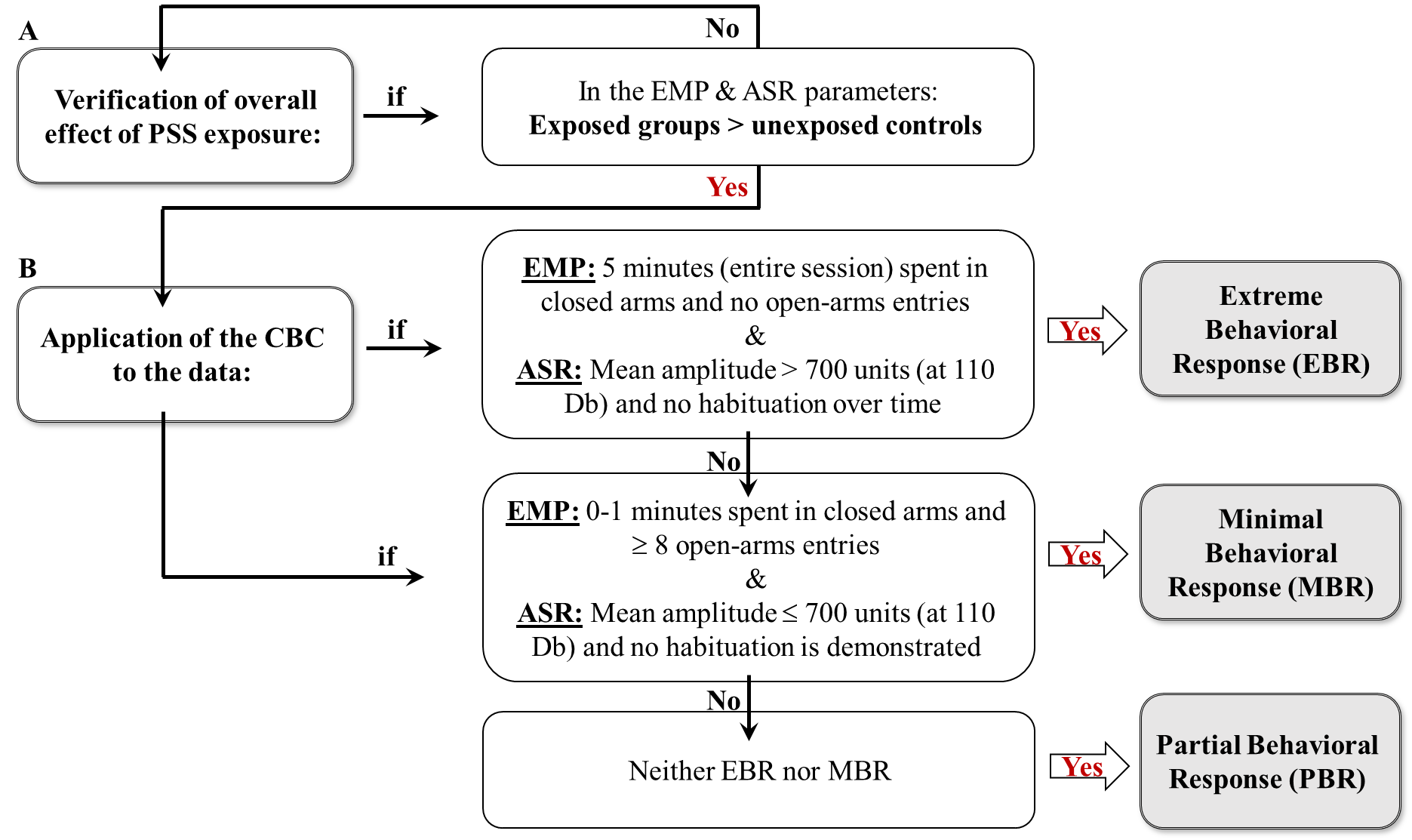
**

**Figure 1: The Cut-off Behavioral Criteria algorithm**. Behavioral models can be more closely matched with contemporary clinical conceptions of PTSD by using an approach that enables the classification of study animals into groups according to the degree of response to a stressor, i.e., the degree to which individual behavior is altered or disrupted.. A) The data must demonstrate that the stressor had a significant effect on the overall behavior of exposed versus non-exposed populations at the time of assessment. B) To maximize the resolution and minimize false positives, extreme responses to both of these paradigms performed in sequence are required for inclusion in the EBR group, whereas a negligible degree of response to both is required for inclusion in the MBR group.

**IMMUNOHISTOCHEMISTRY**

Animals were deeply anesthetized and perfused transcardially with cold 0.9% physiological saline followed by 4% paraformaldehyde (Sigma-Aldrich) in 0.1 M phosphate buffer. Brains were quickly removed, postfixed in the same fixative for 12 h at 4 °C, cryoprotected overnight (30% sucrose in 0.1 M phosphate buffer at 4 °C), and then frozen and stored at -80 °C. Serial coronal sections (20 μm) were obtained with a cryostat (Leica CM 1850; Leica Microsystems, Wetzlar, Germany) and mounted on coated slides.

**Single-label immunocytochemistry:** Sections were air-dried and washed three times in phosphate-buffered saline (PBS) containing Tween 20 (PBS/T) (Sigma-Aldrich). They were then incubated for 60 min in a blocking solution (normal goat serum in PBS) and then overnight at 4 °C with the primary antibodies against c-Fos (rabbit monoclonal anti-c-Fos antiserum [1:1000] product code: sc-166940, Santa Cruz Biotechnology), ORX-A and ORX-B (rabbit polyclonal anti-ORX-A antiserum [1:500], product code: ab-6214, and rabbit polyclonal anti-ORX-B antiserum [1:500], product code: ab-170999, Abcam, Cambridge, UK), NPY (mouse monoclonal anti-NPY antiserum (1:500), product code: sc-133080, Santa Cruz Biotechnology, Inc., Heidelberg, Germany), and BDNF (rabbit polyclonal anti-BDNF antiserum (1:300), product code: sc-ANT-010, Alomone Labs, Jerusalem, Israel). After three washes in PBS/T, the sections were incubated for 2 h in DyLight-488-labeled goat-anti-rabbit IgG or in Dylight-594 goat anti-mouse IgG (1:250; KPL, Gaithersburg, MD, USA) in PBS containing 2% normal goat or horse serum. The sections were subsequently washed and mounted with a mounting medium (Vectastain; Vector Laboratories, Burlingame, CA, USA). Sections from the brains of different groups of rats were processed at the same time and under identical conditions to ensure reliable comparisons and to maintain stringency in tissue preparation and staining conditions. Control staining was performed in the absence of the primary antibodies. Additionally, secondary fluorescent labels were swapped to test for cross-reactivity, and sections were incubated without primary antibodies to test for non-specific binding of the secondary antibodies.

*Relative quantitative analysis of ORX-A- and ORX-B-ir*: Brain sections where ORX-A and ORX-B-ir were found in the PVN (–1.4 to –2.4 mm) and LH (–2.4 to –3.0 mm) were subjected to image analysis. In addition, brain sections where NPY- and BDNF-ir were found in the PVN were subjected to image analysis. Each brain region was defined under the microscope according to cytoarchitectural landmarks ^4^. ORX-ir were measured in a 50,000 μm^2^ area in each region of interest (ROI) and were digitized by using microscopic images (Leica microscope DM4500B) and a DFC340FX digital imaging camera (Leica). Measurements were recorded from predetermined fields in each subregion from both brain hemispheres. The density of fibers and cells expressing ORX-ir, NPY-ir and BDNF (cells) in each area was determined with Leica LAS software (version 3.8). To compensate for background staining levels and control for variations in the overall illumination levels between images, the average pixel density of two regions that presumably contained only nonspecific staining (that is, in areas that are near each ROI that is not thought to contain ORXs) was determined within each captured image, and this value was subtracted from all density measurements performed on that image. c-Fos positive cells were counted manually using a 20 objective and an ocular grid to avoid double counting. Consistent with published studies (Oshitari et al., 2014; Yokoyama et al., 2013), c-Fos neurons were determined only when clear immunostained nuclei were co-localized with DAPI staining.

An additional double-label immunohistochemistry study evaluated the expression pattern of the ORX-A/B-ir cells and fibers within the PVN and LH and in relation to c-Fos neurons.

**Double-label immunohistochemistry of orexins and c-Fos:** Another series of sections from the same cases studied in c-Fos expression experiments were processed for double-labeling for either c-Fos/ORX-A or c-Fos/ORX-B. After processed for c-[Fos protein](https://www.sciencedirect.com/topics/neuroscience/protein-fos) as described above, sections were incubated overnight with primary antibodies against c-Fos (mouse monoclonal anti-c-Fos antiserum [1:250] product code: sc-271243, Santa Cruz Biotechnology), ORX-A and ORX-B (rabbit polyclonal anti-ORX-A antiserum [1:500], product code: ab-6214, and rabbit polyclonal anti-ORX-B antiserum [1:500], product code: ab-170999, Abcam, Cambridge, UK. After three washes in PBS/T, the sections were incubated for 2 h in DyLight-488-labeled goat-anti-rabbit IgG for the detection of ORXs neurons and in Dylight-594 goat anti-mouse IgG for the detection of c-Fos (1:250; KPL, Gaithersburg, MD, USA) in PBS containing 2% normal goat and horse serum. The sections were then washed three times in PBS and examined under a [fluorescence microscope](https://www.sciencedirect.com/topics/biochemistry-genetics-and-molecular-biology/fluorescence-microscope).

After [immunofluorescence](https://www.sciencedirect.com/topics/neuroscience/immunofluorescence) staining, a researcher blinded to the experiment grouping performed counting for the orexin single-labeled neurons and c-Fos + orexin double-labeled neurons. Cells in the PVN and LH exhibiting both c-Fos and orexin immunoreactivity were
counted manually, along with the total number of orexin immunoreactive cells. These numbers were used to calculate the percentage of orexin cells exhibiting c-Fos expression.

1. Cohen H, Matar MA, Joseph Z. Animal models of post-traumatic stress disorder. *Curr Protoc Neurosci* 2013; **Chapter 9:** Unit 9 45.

2. Cohen H, Zohar J, Matar M. The relevance of differential response to trauma in an animal model of posttraumatic stress disorder. *Biol Psychiatry* 2003; **53**(6)**:** 463-473.

3. Cohen H, Zohar J, Matar MA, Kaplan Z, Geva AB. Unsupervised fuzzy clustering analysis supports behavioral cutoff criteria in an animal model of posttraumatic stress disorder. *Biol Psychiatry* 2005; **58**(8)**:** 640-650.

4. Paxinos G, Watson C. *The Rat Brain in Stereotaxic Coordinates*: London, 1988.
